# Prevalence and predictors of diabetes distress and depression in people with type 1 diabetes

**DOI:** 10.3389/fpsyt.2024.1367876

**Published:** 2024-03-22

**Authors:** Abdullah AlOzairi, Mohammad Irshad, Jumana AlKandari, Husain AlSaraf, Ebaa Al-Ozairi

**Affiliations:** ^1^ Department of Psychological Medicine, Faculty of Medicine, Kuwait University, Kuwait City, Kuwait; ^2^ Amiri Hospital, Ministry of Health, Kuwait City, Kuwait; ^3^ DAFNE Unit, Dasman Diabetes Institute, Kuwait City, Kuwait

**Keywords:** type 1 diabetes, diabetes distress, depression, glycemic levels, microvascular complication, lipohypertrophy

## Abstract

**Background:**

People living with diabetes often encounter psychosocial challenges, including diabetes distress and depression. Despite this, little research has focused on the co-occurrence of these conditions. This study aimed to explore the prevalence of depressive symptoms and diabetes distress in people with type 1 diabetes in Kuwait and to identify clinical and demographic factors associated with these conditions.

**Methods:**

A total of 832 people with type 1 diabetes (females: 54.1%, mean age: 29 ± 8.5 years), were invited to participate in Dose Adjustment for Normal Eating (DAFNE) course. Diabetes distress was measured using the Problem Areas in Diabetes (PAID) scale and depressive symptoms were measured using the Patient Health Questionnaire-9 (PHQ-9). Depressive symptoms were defined as PHQ-9 scores ≥10. Data on biomedical outcomes, lifestyle factors, and sociodemographic information were collected.

**Results:**

The prevalence rates of diabetes distress and depressive symptoms were 27.8% and 38.3%, respectively. Notably, 19.6% of people experienced both conditions. In the regression analysis, PAID scale and PHQ-9 scores were significantly associated, patients with higher score on depressive symptoms scale were more likely to suffer diabetes distress (B= 2.65, p < 0.001). Female sex (odds ratio [OR]= 2.2, 95% CI= 1.5, 3.2), higher hemoglobin A1c levels (OR= 1.6, 95% CI= 1.0, 2.5), obesity (OR= 1.7, 95% CI= 1.1, 2.8), inactivity (OR= 2.4, 95% CI= 1.6, 3.6), microvascular complications (OR= 2.8, 95% CI= 1.5, 5.4), and lipohypertrophy (OR= 1.7, 95% CI= 1.1, 2.5) were associated with greater odds for the co-occurrence of diabetes distress and depressive symptoms (p< 0.05 for all).

**Conclusion:**

The majority of people with type 1 diabetes in Kuwait experience both diabetes distress and depressive symptoms. The strong correlation between diabetes distress and depressive symptoms suggests mutual predictability. The co-occurrence of both symptoms is associated with many sociodemographic and clinical factors.

## 1 Introduction

Approximately 73 million adults in the Middle East and North Africa regions are affected by diabetes (1). In Kuwait, approximately 25% of the population live with diabetes (1). The prevalence of type 1 diabetes varies between countries, specifically in the Arab countries, 5-10% of people with diabetes have type 1 diabetes (2).

Diabetes distress encompasses the negative psychological experiences and the challenges of self-management faced by individuals with diabetes. The term is used to describe the despondency and emotional difficulty uniquely associated with diabetes management, such as the constant need for monitoring and insulin dosing, carbohydrate counting, ongoing worries about potential complications, and the risk of deteriorating personal and professional relationships (3, 4). This distress is considered one of the most important psychological concerns in people with type 1 diabetes (5). It is estimated to affect approximately one third of those with diabetes (6). Independently, diabetes-related distress and depression act as obstacles to maintaining diabetes self-care practices. As a result, they lead to loss of control over health indicators (7, 8).

Depression is a common and serious mood disorder in people with diabetes that can cause a persistent feeling of sadness, loss of interest, low self-esteem, fatigue, feelings of worthlessness, and other emotional problems (9, 10). Moreover, major depressive disorder, also known as clinical depression, can significantly affect daily functioning and treatment outcomes (11, 12). Similarly, diabetes distress is a natural emotional response to the demanding self-management of diabetes, including frustration with treatment requirements, concern regarding potential complications, a sense of defeat or hopelessness regarding diabetes management, and reduced motivation for self-care (13). A study suggested that diabetes-distress and depression in people with type 2 diabetes are correlated and overlapping constructs but are not interchangeable (12). Research has linked both depression and diabetes distress to worsened health outcomes, including raised HbA1c levels, elevated diastolic blood pressure, and heightened levels of low-density lipoprotein cholesterol (14, 15). Individuals with depression or diabetes distress are at risk of developing microvascular complications, such as retinopathy, neuropathy, and nephropathy (16). Moreover, those exhibiting higher degrees of DD have been observed to possess a 1.8 times greater risk of premature death and a 1.7 times increased likelihood of developing cardiovascular disease (17), alongside experiencing a diminished quality of life (18) and an increased risk of mortality (7).

Aiming to enhance psychosocial well-being, the American Diabetes Association established recommendations for integrating psychosocial support into patient-centered medical care for all people with diabetes (19). A recent systematic review and meta-analysis reported that the e-health interventions were effective in diminishing diabetes distress among patients with type 2 diabetes (20). In addition, numerous studies also documented the importance of routine assessment of depressive symptoms and diabetic distress in all patients with diabetes (21). However, there is a lack of studies examining the co-occurrence of depressive symptoms and diabetes distress in Arab people with type 1 diabetes. Studies conducted in Western countries have shown that high glucose levels, poor self-management behavior, and lower quality of life are linked to the development of depressive symptoms and diabetic distress (22). More than half of preadolescents with type 1 diabetes reported experiencing either depression or diabetes distress and those who reported higher levels of diabetes distress were also more likely to develop depressive symptoms (22). Further research demonstrated that adolescents with depressive symptoms were four times more likely to experience diabetes distress, indicating that these two challenges frequently co-exist in this group (23). Most research performed in Arab countries has been focused on the psychological wellbeing of people with type 2 diabetes (8, 24); thus, there is a gap of knowledge related to depressive symptoms and disease-related distress in the Arab population with people with type 1 diabetes. Therefore, the aim of this study was to explore the prevalence of depression and diabetes distress in people with type 1 diabetes and identify clinical and demographic factors associated with these conditions. Early recognition, regular screening, and evidence-based treatments for depressive symptoms and diabetes distress can enhance the control over poor health indices, leading to improvement in overall health (25, 26).

## 2 Materials and methods

### 2.1 Study design and setting

This was a cross-sectional study, conducted at the Dasman Diabetes Institute (Dasman, Kuwait). Ethical approval was obtained from the Ministry of Health Kuwait, following the principle of the Declaration of Helsinki. All participants provided written informed consent.

### 2.2 Participants

The study sample comprised individuals with type 1 diabetes invited to participate in the Dose Adjustment for Normal Eating (DAFNE) structured education course at Dasman Diabetes Institute. Individuals meeting the eligibility criteria were invited to participate voluntarily, ensuring anonymity and confidentiality. The inclusion criteria were individuals diagnosed with type 1 diabetes for at least 1 year; aged 18 years and older; and residing in Kuwait. The exclusion criteria included pregnancy, psychosis, dementia, prior participation in any structured lifestyle program designed to cope with diabetes, and inability to communicate in Arabic and/or English.

### 2.3 Data collection

Demographic variables (sex, age, onset of diabetes, nationality, occupation, marital status, smoking status, alcohol consumption, and physical activity) and clinical complications were collected through participant interviews. Body weight (kg), height (measured to the closest 0.5 cm), and waist circumference (cm) were measured, and body mass index (BMI) calculated as Kg/m^2^. The glycemic level (HbA1c%), total cholesterol (mmol/L), high-density lipid cholesterol (mmol/L), low-density lipid cholesterol (mmol/L), and triglycerides (mmol/L) were measured at Dasman Diabetes Institute using the standard procedures. Total cholesterol levels >4 mmol/L, low-density lipid cholesterol levels >2.0 mmol/L, or the use of cholesterol-lowering medication denoted dyslipidemia. Blood pressure was measured over the brachial artery twice, and the diagnosis of hypertension was based on systolic blood pressure ≥130 mmHg and/or diastolic blood pressure ≥80 mmHg on two separate occasions and/or the use of antihypertensive medication. Diabetic complications, such as retinopathy, nephropathy, and cardiovascular disease, were assessed through an interview and the patient record file.

### 2.4 Assessment of depression

The Patient Health Questionnaire-9 (PHQ-9), which comprises a nine-question depression scale, was used to screen for the presence and severity of depressive symptoms according to the Diagnostic and Statistical Manual of Mental Disorders–Fourth Edition, over the past 2 weeks. Each of the nine symptoms was scored using a four-point scale as follows: “0” (not at all); “1” (several days); “2” (more than half the days); or “3” (nearly every day). The total scores ranged from 0 to 27, with higher scores indicating greater severity. A PHQ-9 score ≥10 denoted the presence of depressive symptoms; this threshold has exhibited satisfactory validity in individuals with diabetes (24). The internal reliability (α) of the PHQ-9 was 0.83 in the current sample. The Arabic translation of the PHQ-9 has been previously validated and is a reliable tool in this setting (24).

### 2.5 Assessment of diabetes distress

The Problem Areas in Diabetes (PAID) scale is a measure of a person’s emotional adjustment in response to living with diabetes. Each of the 20 questions corresponds to a potential problem of living with diabetes (e.g., “feeling constantly concerned about food and eating”) and is rated using the following five-point scale: “0” (not a problem); “1” (minor problem); “2” (moderate problem); “3” (somewhat serious problem); and “4” (serious problem). The final PAID scale score is calculated by summing the scores of all 20 questions and multiplying the value by 1.25. The minimum (i.e., 0) and maximum (i.e., 100) scores indicate no and significant diabetes-related distress, respectively. A score >40 denotes clinically significant psychological distress (27). The internal reliability of the PAID scale was optimal (α= 0.938) in the current sample. The Arabic translation of PAID scale has been previously validated in this setting (24).

### 2.6 Statistical analysis

Data were analyzed using IBM SPSS Statistics (version 29.0; IBM Corp., Armonk, NY, USA). The study characteristics were described using the mean ± standard deviation (SD) or proportions (%). The association of depressive symptoms or diabetes distress with participant characteristics was evaluated using logistic regression analysis to obtain an odds ratio (OR) with a 95% confidence interval for each categorical variable, adjusted for sex, age, and diabetes duration. In addition, linear regression analysis was performed to explore the association between the continuous variables. In all analyses, p-values ≤0.05 denoted statistical significance.

## 3 Results

A total of 832 people with type 1 diabetes were interviewed. The demographic and clinical characteristics of all participants are presented in **Table 1**. The mean ± SD age was 29.0 ± 8.5 years, duration of type 1 diabetes was 13.5 ± 8.4 years, hemoglobin A1c (HbA1c) value was 8.5% ± 1.7%, and BMI was 26.3 ± 4.7 kg/m^2^. The proportions of male and female participants were nearly equal (45.9% vs. 54.1%, respectively), with the majority being single (54.7%), employed (55.6%), and of Kuwaiti nationality (88.8%). The participants also had retinopathy (14.4%), neuropathy (4.7%), nephropathy (1.2%), hypertension (12.7%), dyslipidemia (73.7%), and lipohypertrophy (59.0%).

**Table 1:** Demographic and clinical characteristics of the participants (n = 832).

Characteristic		n (%) or Mean (SD)
Sex	Male	382 (45.9)
	Female	450 (54.1)
Age (years)		29.0 (8.5)
Age of onset (years)		15.2 (9.0)
Duration (years)		13.5 (8.4)
Nationality	Kuwaiti	739 (88.8)
	Non-Kuwaiti	93 (11.2)
Marital status	Single	455 (54.7)
	Married	292 (35.1)
	Divorced/widowed	83 (10.0)
Employment status	Employed	454 (54.6)
	Students	222 (26.7)
	Unemployed	52 (6.3)
Exercise status	Active	381 (45.8)
	Sedentary	386 (46.4)
Smoking status	Ex-/Nonsmoker	596 (71.6)
	Current smoker	224 (26.9)
Waist circumference (cm)		85.7 (13.3)
Systolic blood pressure (mmHg)		118.6 (12.7)
Diastolic blood pressure (mmHg)		72.7 (9.4)
BMI (kg/m^2^)		26.3 (4.7)
Normal weight	<25 kg/m^2^	344 (41.3)
Overweight	25–29.9 kg/m^2^	313 (37.6)
Obesity	>29.9 kg/m^2^	175 (21.0)
HbA1c (%)		8.5 (1.7)
HbA1c categories	≥7.5%	613 (73.7)
	<7.5%	219 (26.3)
Cholesterol (mmol/L)		4.8 (0.9)
LDL (mmol/L)		2.9 (0.8)
HDL (mmol/L)		0.8 (0.5)
Triglycerides (mmol/L)		1.5 (0.4)
Macrovascular	Yes	149 (17.9)
Other complications	Yes	517 (62.1)
Neuropathy	Yes	39 (4.7)
Retinopathy	Yes	120 (14.4)
Nephropathy	Yes	10 (1.2)
Hypertension	Yes	106 (12.7)
Dyslipidemia	Yes	613 (73.7)
Lipohypertrophy	Yes	491 (59.0)
PHQ-9 score		7.1 (5.0)
PAID scale score		35.4 (22.5)
Diabetes distress	Yes	319 (38.3)
	No	513 (61.7)
Depressive symptoms	Yes	231 (27.8)
	No	601 (72.2)
Diabetes distress and depressive symptoms		163 (19.6)
Diabetes distress only		156 (18.8)
Depressive symptoms only		68 (8.2)
None		445 (53.5)

SD, Standard deviation.

The mean PHQ-9 scores were positively associated with HbA1c (B = 0.21, p = 0.048), BMI (B = 0.08, p = 0.021), triglycerides (B = 0.64, p = 0.045), and PAID scale scores (B = 0.13, p < 0.001). The PAID scale score was positively correlated with HbA1c (B = 1.08, p = 0.022), BMI (B = 0.33, p = 0.049), triglycerides (B = 3.71, p = 0.01), age (B = 0.28, p = 0.002), and PHQ-9 scores (B = 2.65, p< 0.001) (**Table 2**).

**Table 2:** The association of depression and diabetes stress scores with health indices.

Predictor	Dependent variable			
	PHQ-9 score B (95% CI)*	p-value	PAID scale score B (95% CI)*	p-value
Age (years)	0.04 (0.00, 0.08)	0.062	0.28 (0.10, 0.46)	0.002
Diabetes duration (years)	0.02 (−0.02, 0.06)	0.376	−0.05 (−0.24, 0.13)	0.565
HbA1c (%)	0.21 (0.00, 0.41)	0.048	1.08 (0.16, 2.00)	0.022
BMI (kg/m^2^)	0.08 (0.01, 0.16)	0.021	0.33 (0.00, 0.65)	0.049
Cholesterol (mmol/L)	0.22 (−0.13, 0.58)	0.220	1.23 (−0.39, 2.85)	0.138
LDL-cholesterol (mmol/L)	−0.09 (−0.51, 0.32)	0.661	−0.07 (−1.95, 1.81)	0.944
Triglycerides (mmol/L)	0.64 (0.01, 1.27)	0.045	3.71 (0.87, 6.56)	0.010
HDL-cholesterol (mmol/L)	0.75 (−0.07, 1.57)	0.071	3.01 (−0.69, 6.70)	0.110
Systolic blood pressure (mmHg)	−0.02 (−0.05, 0.01)	0.158	−0.03 (−0.15, 0.09)	0.618
Diastolic blood pressure (mmHg)	−0.01 (−0.05, 0.03)	0.553	0.08 (−0.08, 0.24)	0.336
PAID scale score	0.13 (0.12, 0.14)	<0.001		
PHQ-9 score			2.65 (2.40, 2.90)	<0.001

*B value with 95% Confidence Interval.

The participants were classified based on the clinical cut-off scores of PHQ-9 and PAID scale; 27.8% and 38.3% were positive for depression and diabetes distress, respectively.

Independent analysis of both psychological measures are presented in **Supplementary Table 1**. The results revealed that diabetes distress was significantly associated with higher odds for female sex (OR= 2.0, 95% CI= 1.5, 2.7), microvascular complications (OR = 1.9, 95% CI= 1.2, 3.1), lipohypertrophy (OR= 1.4, 95% CI= 1.0, 1.9), inactivity (OR = 1.8, 95% CI= 1.4, 2.5), and being married (OR = 1.5, 95% CI= 1.0, 2.1) (p< 0.05 for all). Similarly, depressive symptoms were significantly associated with higher odds for female sex (OR= 1.6, 95% CI= 1.2, 2.2), microvascular complications (OR = 2.2, 95% CI= 1.3, 3.7), inactivity (OR= 1.8, 95% CI= 1.3, 2.5), and smoking behavior (OR= 2.2, 95% CI= 1.5, 3.4) (p< 0.05 for all).

**Figure 1:**
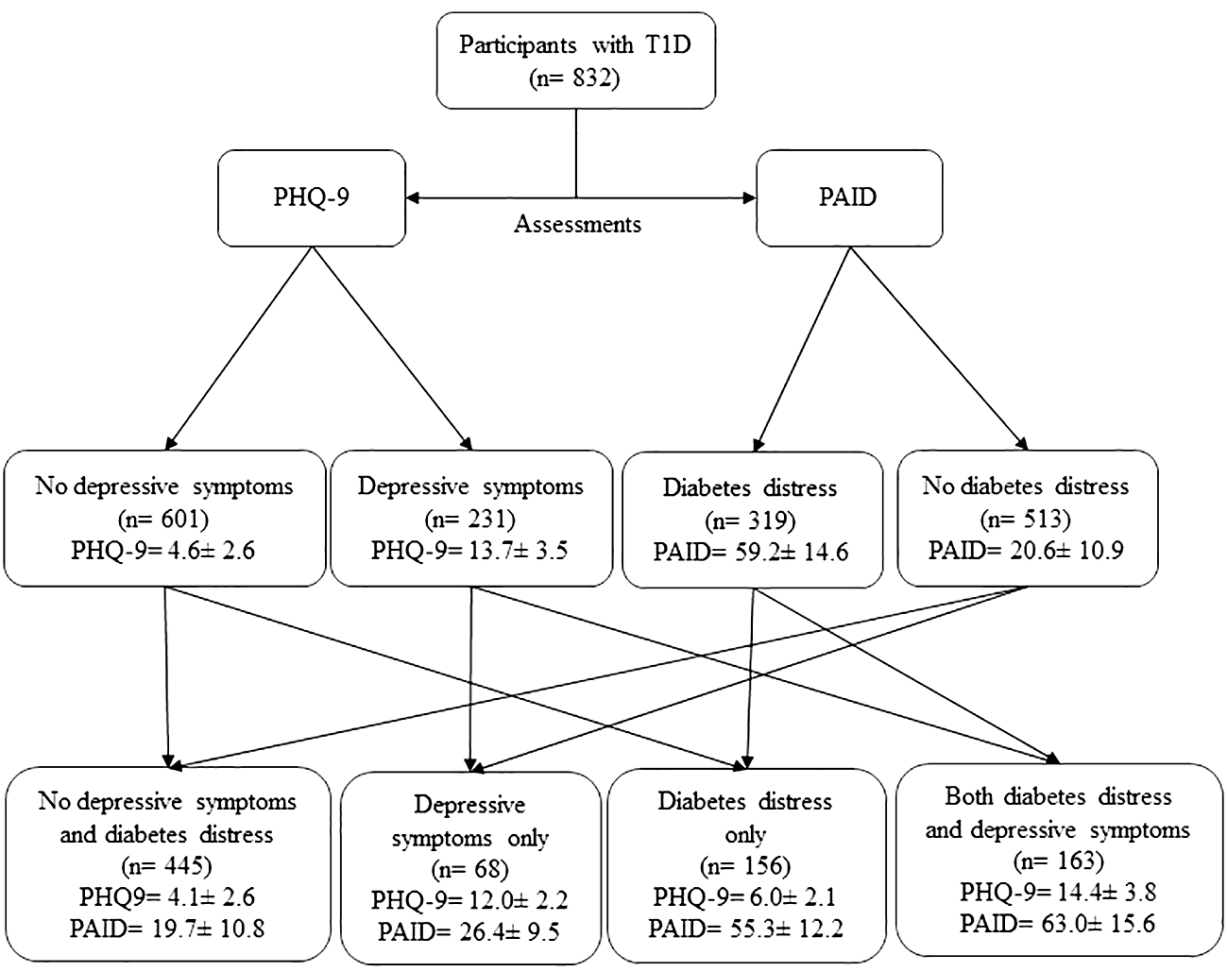
CONSORT flow diagram of diabetes distress and depressive symptoms assessment in people with type 1 diabetes (T1D). PHQ-9 and PAID scores shown in Mean ± Standard deviation.

Participants were further classified based on the co-occurrence of depressive symptoms and diabetes distress into four categories: (1) no presence of depressive symptoms or diabetes distress, 53.5%; (2) depressive symptoms without diabetes distress, 8.2%; (3) diabetes distress without depressive symptoms, 18.8%; and (4) co-occurrence of depressive symptoms and diabetes distress, 19.6%. The mean ± SD of PHQ-9 and PAID scale scores for each category are presented in **Figure 1**.

Through this analysis, we found associations of demographic and clinical factors with depressive symptoms and diabetes distress (**Table 3**). The participants with depressive symptoms but without diabetes distress showed a significantly higher odds for microvascular complications (OR = 2.7, 95% CI= 1.2, 6.5) and smoking behavior (OR = 2.6, 95% CI= 1.3, 5.2). Further, participants with diabetes distress but without depressive symptoms demonstrated significantly higher odds for female sex (OR = 2.0, 95% CI= 1.4, 2.9), microvascular complications (OR = 1.9, 95% CI= 1.0, 3.6), inactivity (OR = 1.5, 95% CI= 1.0, 2.2), and being married (OR = 1.8, 95% CI= 1.2, 2.9) (p< 0.05 for all) compared to those without depressive symptoms and diabetes distress.

**Table 3:** Predictors of depressive symptoms and diabetes stress (diabetes distress and depressive symptoms considered together).

	Co-occurrence of diabetes distress and depressive symptoms (n = 163) †	p-value	Diabetes distress only (n= 156) †	p-value	Depressive symptoms only (n=68) †	p-value
	OR (95% CI) *		OR (95% CI) *		OR (95% CI) *	
Male	1		1		1	
Female	2.2 (1.5, 3.2)	<0.001	2.0 (1.4, 2.9)	<0.001	1.5 (0.9, 2.5)	0.130
HbA1c (<7.5%)	1		1		1	
HbA1c (≥7.5%)	1.6 (1.0, 2.5)	0.043	1.1 (0.7, 1.6)	0.730	1.0 (0.5, 1.7)	0.883
Normal BMI	1		1		1	
Obese BMI	1.7 (1.1, 2.8)	0.029	1.1 (0.6, 1.9)	0.758	1.1 (0.6, 2.2)	0.769
Overweight BMI	0.9 (0.6, 1.3)	0.529	1.3 (0.8, 1.9)	0.243	0.6 (0.3, 1.1)	0.089
Complication (no)	1		1		1	
Microvascular	2.8 (1.5, 5.4)	0.001	1.9 (1.0, 3.6)	0.043	2.7 (1.2, 6.1)	0.020
Other complications	1.0 (0.6, 1.6)	0.985	0.7 (0.4, 1.1)	0.160	0.7 (0.4, 1.4)	0.350
Lipohypertrophy (no)	1		1		1	
Lipohypertrophy (yes)	1.7 (1.1, 2.5)	0.010	1.1 (0.8, 1.7)	0.518	0.9 (0.5, 1.6)	0.791
Students	1		1		1	
Employed	1.0 (0.6, 1.8)	0.881	1.2 (0.7, 2.0)	0.527	1.7 (0.8, 3.5)	0.157
Unemployed	0.8 (0.3, 2.0)	0.637	1.1 (0.5, 2.6)	0.815	2.7 (1.0, 7.6)	0.060
Physically active	1		1		1	
Inactive (sedentary)	2.4 (1.6, 3.6)	<0.001	1.5 (1.0, 2.2)	0.051	1.3 (0.7, 2.2)	0.376
Marital status- Single	1		1		1	
Divorced	0.6 (0.3, 1.2)	0.121	1.0 (0.5, 1.9)	0.907	0.9 (0.4, 2.2)	0.793
Married	1.1 (0.7, 1.8)	0.559	1.8 (1.1, 2.9)	0.016	0.8 (0.4, 1.7)	0.577
Non-smoking	1		1		1	
Current smoker	2.2 (1.3, 3.6)	0.003	1.1 (0.7, 1.8)	0.719	2.6 (1.3, 5.2)	0.006
Nationality- Kuwaiti	1		1		1.0	
Non-Kuwaiti	1.3 (0.7, 2.3)	0.349	1.2 (0.7, 2.2)	0.444	0.9 (0.4, 2.2)	0.787
Hypertension (no)	1		1		1	
Hypertension (yes)	1.0 (0.6, 1.8)	0.988	1.2 (0.7, 2.1)	0.556	0.5 (0.2, 1.4)	0.174
Dyslipidemia (no)	1		1		1	
Dyslipidemia (yes)	0.9 (0.6, 1.4)	0.759	0.7 (0.5, 1.1)	0.155	0.9 (0.5, 1.6)	0.670

† Refence category is the participants without diabetes distress and depressive symptoms in our sample.

* Adjusted for sex, age, and diabetes duration for all variables except for sex, which was adjusted for age and diabetes duration.

OR (95% CI), Odd ratio with 95% Confidence Interval.

In addition, the individuals experiencing both depressive symptoms and diabetes distress together had a significantly higher odds for female sex (OR = 2.2, 95% CI= 1.5, 3.2), elevated glycemic levels (OR= 1.6, 95% CI= 1.1, 2.5), obesity (OR = 1.7, 95% CI= 1.1, 2.8), microvascular complications (OR = 2.8, 95% CI= 1.5, 5.4), lipohypertrophy (OR = 1.7, 95% CI= 1.1, 2.5), inactivity (OR = 2.4, 95% CI= 1.6, 3.6) and smoking behavior (OR = 2.2, 95% CI= 1.3, 3.6) (p< 0.05 for all).

**Figure 2:**
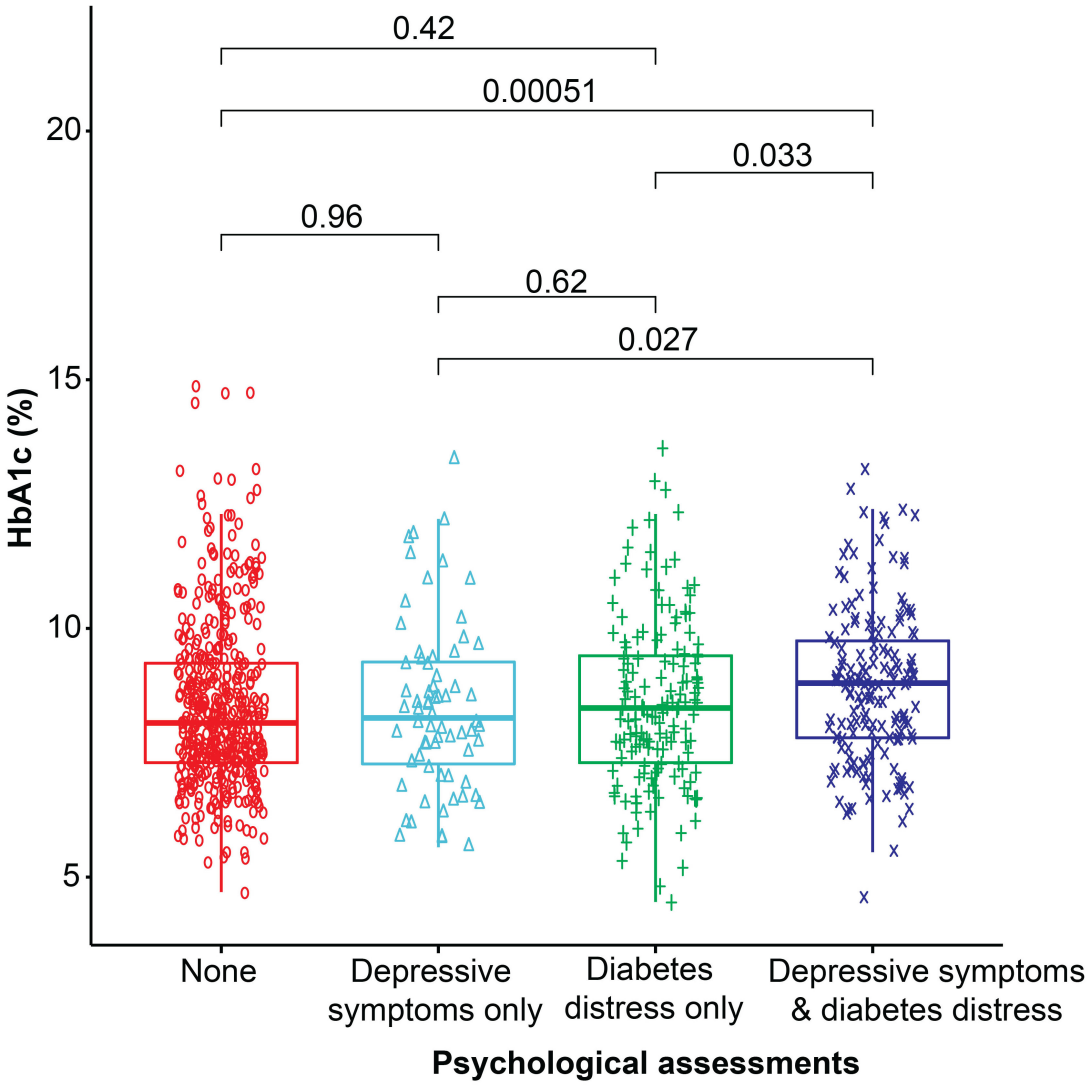
Box plot representing the median glycemic levels (HbA1c %) in each category. The Wilcoxon test was used to compare the two groups.

The co-occurrence of diabetes distress and depressive symptoms in female participants showed a greater odds ratio for higher glycemic levels (OR= 1.9, 95% CI= 1.0, 3.4), obesity (OR= 2.0, 95% CI= 1.0, 3.7), microvascular complications (OR= 4.1, 95% CI= 1.7, 10.1), and lipohypertrophy (OR= 2.1, 95% CI= 1.2, 3.5) (p< 0.05 for all), compared to their male counterparts. However, inactivity associated with the co-occurrence of these symptoms was almost equal in male and female participants (**Supplementary Table 2**). The glycemic levels for each group of participants are presented in **Figure 2**.

## 4 Discussion

This is the first study that simultaneously explored the two common mental health concerns associated with type 1 diabetes in a large sample of people in the Arab region. Mental health concerns were assessed using two distinct psychological measurement scales, and both scales demonstrated optimal internal validity in the current sample. We found that a significant number of people with type 1 diabetes experienced both diabetes distress and depressive symptoms. People experiencing these conditions were likely to be female, and have microvascular complications, lipohypertrophy, higher glycemic levels, obesity, and a sedentary lifestyle. In the present study, 20% of participants experienced both diabetes distress and depressive symptoms, while around 19% experienced diabetes distress without depression and 8% experienced depression without diabetes distress. There is limited information available on the co-occurrence of diabetes-related distress and depression in people with diabetes. A study reported that among African American adults with type 2 diabetes, 17.8% experienced both diabetes-related distress and depression, 17.4% experienced diabetes-related distress without depression, and 7.3% experienced depression without diabetes-related distress (28). Another study reported that the co-occurrence of clinical depression and diabetes-related distress among people with both types of diabetes was 6.1%, diabetes-related distress without depression was 17.8% and depression without diabetes-related distress was 5.7% (29). In our study, the co-occurrence of diabetes distress and depression was slightly higher than in these other studies, possibly due to differences in the type of diabetes being studied.

In the present study, a significant overlap was observed between depressive symptoms and diabetes distress. Specifically, seven of 10 participants with depressive symptoms also experienced diabetes distress. Similarly, five of 10 individuals with diabetes distress also experienced depressive symptoms. The results suggested that the relationship between diabetes distress and depression is often bidirectional, underlining the complex interplay between the physical and emotional aspects of diabetes management. The stress and emotional burden associated with the management of diabetes may contribute to the development or exacerbation of depression. In turn, depression can further complicate the management of diabetes.

In the present study, 38.3% participants scored above the cut-point for diabetes distress, which is higher than that reported in our previous study in people with type 2 diabetes, where only 14% had diabetes distress (24). However, the proportion of participants scoring above the cut point for depressive symptoms in the present study was 27.8%, which is nearly equal to that recorded in people with type 2 diabetes in Kuwait (24). The high prevalence of diabetes distress in people with type 1 diabetes compared to our previous study in type 2 diabetes could be attributed to several factors. For instance, it is plausible that the lower mean age of people with type 1 diabetes versus type 2 diabetes (29.0 ± 8.5 vs. 55.3 ± 10.1 years, respectively) and the constant need for exogenous insulin treatment may have contributed to the higher prevalence of diabetes distress in people with type 1 diabetes. People with type 2 diabetes who received insulin treatment were at a higher risk for developing diabetes distress than those who had only oral medication (24). Another possibility is that the source of diabetes distress in people with type 1 and type 2 diabetes may differ. For instance, lipohypertrophy (reported by almost 60% of people with type 1 diabetes) may be one of the factors responsible for the development of diabetes distress. Patients with lipohypertrophy had higher scores for depression and diabetes distress versus those without (data not shown). A study reported that people with type 1 diabetes and more lipohypertrophy areas had higher scores for depression and lower scores for treatment satisfaction with regard to glycemic control (30).

Most studies have reported on either diabetes distress or depression in people with type 1 diabetes. Recent studies from Norway have shown elevated levels of diabetes distress in 21.7% of people with type 1 diabetes (31), while a study from Croatia showed elevated level of diabetes distress in 36% of people with type 1 diabetes (32). Compared to both studies, the present study showed a high diabetes distress rate using the same cut-point. Similarly, the prevalence of depressive symptoms in the present study was higher than that reported from developing countries using the same cut-point (33). The prevalence rate of diabetes distress and depressive symptoms differs across studies possibly due to differences in study populations and diabetes care settings.

The presence of severe diabetes distress can predispose individuals to problematic self-care behavior (34). It is likely that this process increases the chance of poor adherence to treatment and the risk of diabetes-related complications (35). In the present study, microvascular complications and sedentary lifestyle may be independent contributory factors to diabetes distress and depressive symptoms. This association is strengthened when considering the co-occurrence of diabetes distress and depressive symptoms together. The presence of lipohypertrophy was likely a contributory factor in those who experienced both diabetes distress and depressive symptoms. However, this association was not found in those who experienced diabetes distress alone or depressive symptoms alone. Similarly, hyperglycemia was a contributing factor in those who experienced both diabetes distress and depressive symptoms, but this association was not found in those who experienced diabetes distress alone or depressive symptoms alone. These results emphasize the importance of concurrent screening for both clinically important psychological conditions.

Depressive symptoms and diabetes distress occur more frequently in females than in males, and this pattern is observed even when considering the co-occurrence of depressive symptoms and diabetes distress. Particularly, female participants experiencing both diabetes distress and depressive symptoms had a higher likelihood of elevated glycemic levels, obesity, microvascular complications, and lipohypertrophy compared to males. These findings suggest that sex differences play a role in the manifestation of these symptoms and their associated complications in people with diabetes. Many international studies have reported that a higher proportion of females with type 1 diabetes had higher levels of diabetes distress (5, 32, 36) and depressive symptoms (37, 38) than males. The sex discrepancy for these psychological measures was also noted in our previous study of people with type 2 diabetes (24). Collectively, these data reflect the trend of elevated diabetes distress and depression among females. A possible explanation for this discrepancy may be that males and females exhibit behavioral differences in the management of diabetes and emotional distress (39). Additionally, females appear to be more affected by diabetes distress in the context of their marital relationships (39).

Studies have reported varying results with regard to the relationship between blood glucose levels and depression or diabetes distress (5, 32). Similar to these studies, our results showed that hyperglycemia was independently associated with depression and diabetes distress scores. Moreover, hyperglycemia was associated with a higher likelihood of co-occurrence of depressive symptoms and diabetes distress. It is plausible that the co-occurrence of depressive symptoms and diabetes distress may exacerbate the mental health problems of patients, thereby potentially affecting glycemic control. Furthermore, uncontrolled hyperglycemia may increase concerns regarding the consequences of diabetes and perceived treatment failures, thus leading to overwhelming feelings of depression and diabetes distress. A study reported that depression and diabetes distress can reduce adherence to self-care practices, which, in turn, may contribute to loss of glycemic control (40).

Smoking behavior was significantly associated with depressive symptoms, as well as the co-occurrence of diabetes distress and depressive symptoms. There is substantial evidence suggesting an association between smoking and increased levels of diabetes distress in people with diabetes (31, 41).. This result underscores the cumulative impact of these two psychological conditions. Physical inactivity is a causal factor for obesity and distress, leading to poor glycemic control and metabolic imbalance (42, 43). Failure to control elevated blood sugar levels over an extended period can lead to retinopathy, nephropathy, neuropathy, and malfunction of vital organs (e.g., heart, blood vessels) (44). Moreover, physical inactivity and depressed mood are associated with a higher likelihood of diabetes-related complications (45–47). Additionally, people with diabetes complications and diabetes distress are at an increased risk of depressive symptoms (48). Severe depressive symptoms further increase the likelihood of poor treatment outcomes and diabetes-related complications (35). These findings underscore the complex interplay between physical inactivity, psychological well-being, and the management of diabetes, thus emphasizing the need for holistic approaches to address these interconnected factors. Efforts targeted at enhancing mental well-being and physical activity are crucial to improve the long-term health of individuals with type 1 diabetes. A study on DAFNE training courses revealed that they have a positive impact on both the biomedical and psychological status of individuals with type 1 diabetes (49). Emphasizing an active lifestyle can play a crucial role in encouraging self-care engagement and enhancing mental well-being (49). Participation in a DAFNE training course can reduce symptoms of depression and diabetes distress, as well as the comorbid risks to physical health, in people with type 1 diabetes in Kuwait.

This study has strengths and limitations. First, it included a substantial number of adults living with type 1 diabetes, thereby enhancing the statistical power of the investigation and the generalizability of findings. Second, the concurrent exploration of diabetes distress and depressive symptoms provides a more holistic understanding of the psychological experiences of individuals with type 1 diabetes. However, the cross-sectional design of this study limits the ability to establish causation or the sequence of events. Additionally, the study did not encompass all sociodemographic factors, such as income or life events, which could potentially influence psychological outcomes. In addition, the participants were treated in specialized secondary care centers; thus, their characteristics may differ from those of people treated in primary care settings.

## 5 Conclusion

The prevalence of both diabetes distress and depressive symptoms is high among people with type 1 diabetes in Kuwait. The co-occurrence of diabetes distress and depressive symptoms is associated with various sociodemographic and clinical factors. Furthermore, higher levels of diabetes distress are strongly correlated with depressive symptoms, indicating that distress and depression may be mutually predictive. The results of this study recommend assessing these constructs in routine clinical practice to identify distress and depressive symptoms that may warrant additional support. This is especially true in the Arab region, where there is limited psychosocial screening, despite this being included in international guidelines.

## Data availability statement

The original contributions presented in the study are included in the article/**Supplementary Material**. Further inquiries can be directed to the corresponding author.

## Ethics statement

The studies involving humans were approved by Ethical committee, Ministry of Health, Kuwait. The studies were conducted in accordance with the local legislation and institutional requirements. The participants provided their written informed consent to participate in this study.

## Author contributions

AA: Supervision, Validation, Visualization, Writing – original draft, Writing – review & editing, Software, Resources, Project administration, Methodology, Investigation, Funding acquisition, Formal Analysis, Data curation, Conceptualization. MI: Conceptualization, Data curation, Formal Analysis, Investigation, Methodology, Software, Supervision, Validation, Writing – original draft, Writing – review & editing. JA: Data curation, Resources, Visualization, Writing – review & editing. HA: Conceptualization, Data curation, Formal Analysis, Investigation, Supervision, Validation, Writing – review & editing. EA-O: Conceptualization, Data curation, Formal Analysis, Funding acquisition, Investigation, Methodology, Project administration, Resources, Software, Supervision, Validation, Visualization, Writing – original draft, Writing – review & editing.
