# Supplementary material for: Prevalence and predictors of diabetes distress and depression in people with type 1 diabetes
Source: Front Psychiatry. 2024 Mar 22;15:1367876. doi: 10.3389/fpsyt.2024.1367876 (PMC10995252; doi:10.3389/fpsyt.2024.1367876)
Supplement: Supplementary file 1 [file Table_1.docx]

**Supplementary Table 1:** Predictors of depressive symptoms and diabetes stress (independent analysis)

|  | Diabetes distress  (n = 319) |  | Depressive symptoms  (n = 231) | |
| --- | --- | --- | --- | --- |
|  | OR (95% CI)*‡ | p-value | OR (95% CI)*† | p-value |
| Male | 1 |  | 1 |  |
| Female | 2.0 (1.5, 2.7) | <0.001 | 1.6 (1.2, 2.2) | 0.002 |
| HbA1c (<7.5%) | 1 |  | 1 |  |
| HbA1c (≥7.5%) | 1.3 (0.9, 1.8) | 0.116 | 1.3 (0.9, 1.9) | 0.125 |
| Normal BMI | 1 |  | 1 |  |
| Obese BMI | 1.4 (0.9, 2.1) | 0.097 | 1.5 (1.0, 2.2) | 0.065 |
| Overweight BMI | 1.1 (0.8, 1.6) | 0.431 | 0.7 (0.5, 1.0) | 0.073 |
| Complication (no) | 1 |  | 1 |  |
| Microvascular | 1.9 (1.2, 3.1) | 0.009 | 2.2 (1.3, 3.7) | 0.003 |
| Other complications | 0.9 (0.6, 1.3) | 0.460 | 1.0 (0.7, 1.5) | 0.929 |
| Lipohypertrophy (no) | 1 |  | 1 |  |
| Lipohypertrophy (yes) | 1.4 (1.0, 1.9) | 0.032 | 1.4 (1.0, 1.9) | 0.070 |
| Students | 1 |  | 1 |  |
| Employed | 1.0 (0.7, 1.6) | 0.881 | 1.1 (0.7, 1.8) | 0.564 |
| Unemployed | 0.8 (0.4, 1.6) | 0.536 | 1.2 (0.6, 2.4) | 0.639 |
| Physically active | 1 |  | 1 |  |
| Inactive (sedentary) | 1.8 (1.4, 2.5) | <0.001 | 1.8 (1.3, 2.5) | <0.001 |
| Marital status- Single | 1 |  | 1 |  |
| Divorced | 0.7 (0.4, 1.3) | 0.293 | 0.7 (0.4, 1.2) | 0.154 |
| Married | 1.5 (1.0, 2.1) | 0.042 | 0.9 (0.6, 1.3) | 0.528 |
| Non-smoker | 1 |  | 1 |  |
| Current smoker | 1.4 (0.9, 2.0) | 0.123 | 2.2 (1.5, 3.4) | <0.001 |
| Nationality- Kuwaiti | 1 |  | 1 |  |
| Non-Kuwaiti | 1.3 (0.8, 2.0) | 0.251 | 1.1 (0.7, 1.8) | 0.691 |
| Hypertension (no) | 1 |  | 1 |  |
| Hypertension (yes) | 1.2 (0.7, 1.8) | 0.506 | 0.8 (0.5, 1.3) | 0.393 |
| Dyslipidemia (no) | 1 |  | 1 |  |
| Dyslipidemia (yes) | 0.8 (0.6, 1.2) | 0.314 | 1.0 (0.7, 1.4) | 0.990 |

* Adjusted for sex, age, and diabetes duration for all variables except for sex, which was adjusted for age and diabetes duration.

†Reference is without diabetes distress.

‡Reference is without depressive symptoms.

**Supplementary Table 2:** Predictors of depressive symptoms and diabetes stress (diabetes distress and depressive symptoms considered together) stratified by the sex.

|  | Male† |  |  |  |  |  | Female‡ |  |  |  |  |  |
| --- | --- | --- | --- | --- | --- | --- | --- | --- | --- | --- | --- | --- |
|  | Co-occurrence of diabetes distress and depressive symptoms  (n = 58) | | Diabetes distress only  (n = 58) | | Depressive symptoms only (n = 29) | | Co-occurrence of diabetes distress and depressive symptoms  (n = 105) | | Diabetes distress only  (n = 98) | | Depressive symptoms only (n = 39) | |
|  | OR (95% CI)* | p-value | OR (95% CI)* | p-value | OR (95% CI)* | p-value | OR (95% CI)* | p-value | OR (95% CI)* | p-value | OR (95% CI)* | p-value |
| HbA1c (<7.5%) | 1 |  | 1 |  | 1 |  | 1 |  | 1 |  | 1 |  |
| HbA1c (≥7.5%) | 1.4 (0.7, 2.6) | 0.371 | 0.7 (0.4, 1.2) | 0.176 | 0.8 (0.4, 1.8) | 0.600 | 1.9 (1.0, 3.4) | 0.043 | 1.6 (0.9, 3.0) | 0.102 | 1.2 (0.5, 2.6) | 0.724 |
| Normal BMI | 1 |  | 1 |  | 1 |  | 1 |  | 1 |  | 1 |  |
| Obese BMI | 1.4 (0.7, 3.1) | 0.335 | 0.6 (0.2, 1.4) | 0.227 | 0.7 (0.3, 2.0) | 0.547 | 2.0 (1.0, 3.7) | 0.039 | 1.7 (0.8, 3.4) | 0.168 | 1.5 (0.6, 4.0) | 0.373 |
| Overweight BMI | 0.7 (0.4, 1.5) | 0.371 | 0.8 (0.4, 1.6) | 0.553 | 0.3 (0.1, 0.9) | 0.032 | 1.0 (0.6, 1.7) | 0.982 | 1.7 (1.0, 2.8) | 0.064 | 0.9 (0.4, 1.9) | 0.690 |
| Complication (no) | 1 |  | 1 |  | 1 |  | 1 |  | 1 |  | 1 |  |
| Microvascular | 2.1 (0.8, 5.2) | 0.131 | 0.8 (0.3, 2.1) | 0.638 | 1.1 (0.3, 3.6) | 0.921 | 4.1 (1.7, 10.1) | 0.002 | 4.0 (1.7, 9.6) | 0.002 | 6.5 (2.0, 20.8) | 0.002 |
| Other complications | 0.7 (0.3, 1.5) | 0.337 | 0.5 (0.2, 1.0) | 0.038 | 0.6 (0.2, 1.4) | 0.219 | 1.3 (0.7, 2.6) | 0.407 | 1.0 (0.5, 1.9) | 0.956 | 1.0 (0.4, 2.5) | 0.970 |
| Lipohypertrophy (no) | 1 |  | 1 |  | 1 |  | 1 |  | 1 |  | 1 |  |
| Lipohypertrophy (yes) | 1.3 (0.7, 2.5) | 0.337 | 0.8 (0.4, 1.4) | 0.439 | 0.9 (0.4, 2.0) | 0.840 | 2.1 (1.2, 3.5) | 0.007 | 1.5 (0.9, 2.5) | 0.119 | 1.0 (0.5, 2.0) | 0.985 |
| Students | 1 |  | 1 |  | 1 |  | 1 |  | 1 |  | 1 |  |
| Employed | 1.1 (0.4, 2.8) | 0.843 | 1.2 (0.5, 3.1) | 0.707 | 1.4 (0.4, 4.4) | 0.564 | 1.0 (0.5, 1.9) | 0.935 | 1.2 (0.6, 2.2) | 0.620 | 1.8 (0.7, 4.8) | 0.214 |
| Unemployed | 0.0 (0.0, 0.0) |  | 1.4 (0.2, 10.0) | 0.719 | 5.2 (1.0, 26.3) | 0.048 | 0.9 (0.4, 2.5) | 0.896 | 1.1 (0.4, 2.9) | 0.845 | 2.1 (0.5, 8.0) | 0.295 |
| Physically active | 1 |  | 1 |  | 1 |  | 1 |  | 1 |  | 1 |  |
| Inactive (sedentary) | 2.5 (1.3, 4.5) | 0.004 | 1.2 (0.7, 2.3) | 0.522 | 1.3 (0.6, 3.0) | 0.505 | 2.4 (1.4, 4.0) | 0.001 | 1.6 (1.0, 2.7) | 0.060 | 1.2 (0.6, 2.6) | 0.557 |
| Marital status- Single | 1 |  | 1 |  | 1 |  | 1 |  | 1 |  | 1 |  |
| Divorced | 0.2 (0.0, 1.1) | 0.064 | 1.0 (0.4, 2.9) | 0.940 | 0.9 (0.3, 3.1) | 0.859 | 0.8 (0.3, 1.9) | 0.603 | 0.9 (0.3, 2.3) | 0.817 | 0.8 (0.2, 3.1) | 0.769 |
| Married | 1.0 (0.5, 2.0) | 0.900 | 1.2 (0.6, 2.6) | 0.589 | 0.5 (0.2, 1.4) | 0.167 | 1.3 (0.7, 2.5) | 0.352 | 2.4 (1.3, 4.6) | 0.006 | 1.3 (0.5, 3.5) | 0.551 |
| Non-smoking | 1 |  | 1 |  | 1 |  | 1 |  | 1 |  | 1 |  |
| Current smoker | 2.1 (1.1, 3.8) | 0.020 | 1.2 (0.7, 2.2) | 0.518 | 2.2 (1.0, 4.9) | 0.062 | 2.2 (0.9, 5.6) | 0.105 | 0.7 (0.2, 2.6) | 0.572 | 3.4 (1.1, 11.0) | 0.038 |
| Nationality- Kuwaiti | 1 |  | 1 |  | 1 |  | 1 |  | 1 |  | 1 |  |
| Non-Kuwaiti | 1.5 (0.6, 3.8) | 0.379 | 2.5 (1.1, 5.7) | 0.027 | 2.4 (0.8, 7.2) | 0.105 | 1.1 (0.6, 2.2) | 0.779 | 0.7 (0.3, 1.6) | 0.391 | 0.2 (0.0, 1.5) | 0.112 |
| Hypertension (no) | 1 |  | 1 |  | 1 |  | 1 |  | 1 |  | 1 |  |
| Hypertension (yes) | 1.0 (0.5, 2.1) | 0.925 | 1.3 (0.6, 2.6) | 0.522 | 0.5 (0.1, 1.8) | 0.308 | 1.0 (0.4, 2.3) | 0.917 | 0.9 (0.3, 2.2) | 0.786 | 0.3 (0.0, 2.7) | 0.309 |
| Dyslipidemia (no) | 1 |  | 1 |  | 1 |  | 1 |  | 1 |  | 1 |  |
| Dyslipidemia (yes) | 0.7 (0.4, 1.4) | 0.302 | 0.6 (0.3, 1.1) | 0.099 | 0.6 (0.3, 1.4) | 0.251 | 1.2 (0.7, 2.0) | 0.589 | 0.9 (0.5, 1.5) | 0.661 | 1.2 (0.5, 2.8) | 0.621 |

*Adjusted for sex, age, and diabetes duration for all variables

†Reference category is the participants without diabetes distress and depressive symptoms in male groups.

‡Reference category is the participants without diabetes distress and depressive symptoms in female groups.
